# EMT‐ and MET‐related processes in nonepithelial tumors: importance for disease progression, prognosis, and therapeutic opportunities

**DOI:** 10.1002/1878-0261.12085

**Published:** 2017-06-19

**Authors:** Ulf D. Kahlert, Justin V. Joseph, Frank A. E. Kruyt

**Affiliations:** ^1^ Department of Neurosurgery Medical Faculty Heinrich‐Heine University Düsseldorf Germany; ^2^ Department of Biomedicine University of Bergen Norway; ^3^ Department of Medical Oncology University of Groningen University Medical Center Groningen The Netherlands

**Keywords:** cancer stem cell, epithelial, glioblastoma, leukemia, mesenchymal, sarcoma

## Abstract

The epithelial‐to mesenchymal (EMT) process is increasingly recognized for playing a key role in the progression, dissemination, and therapy resistance of epithelial tumors. Accumulating evidence suggests that EMT inducers also lead to a gain in mesenchymal properties and promote malignancy of nonepithelial tumors. In this review, we present and discuss current findings, illustrating the importance of EMT inducers in tumors originating from nonepithelial/mesenchymal tissues, including brain tumors, hematopoietic malignancies, and sarcomas. Among these tumors, the involvement of mesenchymal transition has been most extensively investigated in glioblastoma, providing proof for cell autonomous and microenvironment‐derived stimuli that provoke EMT‐like processes that regulate stem cell, invasive, and immunogenic properties as well as therapy resistance. The involvement of prominent EMT transcription factor families, such as TWIST, SNAI, and ZEB, in promoting therapy resistance and tumor aggressiveness has also been reported in lymphomas, leukemias, and sarcomas. A reverse process, resembling mesenchymal‐to‐epithelial transition (MET), seems particularly relevant for sarcomas, where (partial) epithelial differentiation is linked to less aggressive tumors and a better patient prognosis. Overall, a hybrid model in which more stable epithelial and mesenchymal intermediates exist likely extends to the biology of tumors originating from sources other than the epithelium. Deeper investigation and understanding of the EMT/MET machinery in nonepithelial tumors will shed light on the pathogenesis of these tumors, potentially paving the way toward the identification of clinically relevant biomarkers for prognosis and future therapeutic targets.

**Abbreviations**
ALK‐ALCL: anaplastic lymphoma kinase–anaplastic large cell lymphoma
ALL: acute lymphoid leukemia
AMF/PGI: autocrine motility factor/phosphoglucose isomerase
AML: acute myeloid leukemia
ATLL: adult T‐cell leukemia/lymphoma
BCL6: B‐cell lymphoma protein 6
BL: Burkitt lymphoma
BM: bone marrow
BMI: B lymphoma Mo‐MLV insertion region 1 homolog
CLL: chronic lymphocytic leukemia
CML: chronic myeloid leukemia
CNS: central nervous system
CSC: cancer stem cell
CTC: circulating tumor cells
CTCL: cutaneous T‐cell lymphoma
DIPG: diffuse intrinsic pontine glioma
E/M: epithelial/mesenchymal
EMT: epithelial‐to‐mesenchymal transition
EMT‐TFs: EMT transcription factors
GBM: glioblastoma
GNP: granule neuron precursors
HL: Hodgkin's lymphoma
MCL: mantle cell lymphoma
MET: mesenchymal‐to‐epithelial transition
MM: multiple myeloma
MMP: matrix metalloproteinase
MT: mesenchymal transition
NHL: non‐Hodgkin's lymphoma
NSC: neural stem cell
RITL: radiation‐induced thymic lymphoma
SVZ: subventricular zone
Sz: Sézary syndrome
TME: tumor microenvironment
TMZ: temozolomide
WFA: withaferin‐A

## 1 Introduction

The ability of epithelial cells to lose their defining epithelial features and gaining a more loosely oriented mesenchymal phenotype is known as the process of epithelial/mesenchymal transition (EMT). EMT has been well described during embryonic development and is an essential process required for the formation of mesoderm and the neural tube (Kalluri and Weinberg, 2009; Thiery *et al*., 2009). In organisms, EMT plays a role in wound healing and tissue fibrosis. Upon EMT, cell polarity and cell–cell adhesion are lost and cells gain a migratory and invasive phenotype that is characteristic for mesenchymal cells.

**Figure 1:**
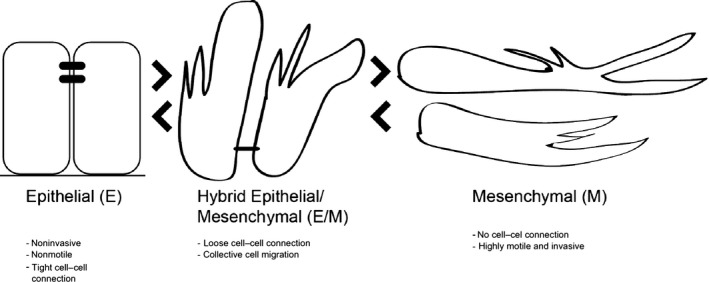
Hybrid and reciprocal phenotypes of EMT/MET. Schematic representation of reciprocal phenotypic conversion of epithelial (E), hybrid epithelial/mesenchymal (E/M), and mesenchymal (M) phenotypes of cancer cells.

In cancer, a key role for EMT in tumor progression has been proposed (Thiery, 2002). Over the last decade, accumulating evidence has indicated an important role for EMT in various malignant properties of tumor cells including tumor infiltration, metastasis, cancer stem cell (CSC) properties, therapy resistance, and immunosuppression (De Craene and Berx, 2013; Yang and Weinberg, 2008; Ye and Weinberg, 2015). In general, the mesenchymal state of tumor cells, characterized by cell autonomous motility and invasiveness, has been associated with worse clinical prognosis. In essence, EMT is a reversible process and cells can revert back to an epithelial state by mesenchymal/epithelial transition (MET) that appears to be essential for colonization of tumor cells at distant sites, a key step in the metastatic process. Of note, cells display plasticity to the EMT/MET processes and may possess intermediate features of these two opposite states (Ye and Weinberg, 2015). In fact, recent reports indicate the existence of cells with a stable hybrid epithelial/mesenchymal (E/M) status simultaneously expressing epithelial and mesenchymal markers. E/M is characterized by weak cell adhesions, migration in multicell aggregates giving rise to circulating tumor cells, enhanced stemness, and therapy resistance (reviewed in Jolly *et al*. 2015). Each of the three phenotypes of EMT, epithelial, hybrid, and mesenchymal, seems to take on distinct responsibilities during cancer progression, indicating the complexity of this process and the challenge to find efficient therapies to comprehensively combat tumor metastasis (for schematic representation, see Fig. 1).

The initiation of EMT and maintenance of a mesenchymal state is controlled by multiple heterotypic signals and is context dependent (Nieto and Cano, 2012). During tumor progression, cell autonomous triggers as well as paracrine signals derived from stromal cells such as fibroblasts and immune cells regulate the EMT status. The cell intrinsic molecular mechanisms of EMT have been examined extensively and consist of complex and overlapping signaling networks. The hallmark of EMT is loss of the cell adhesion glycoprotein E‐cadherin, encoded by the *CDH1* gene, leading to the loss of intercellular junctions/cell–cell interactions and alterations in intermediate filament composition from cytokeratins to vimentin, allowing cells to dissociate and gain migratory potential. The enhanced secretion of proteases, particularly the matrix metalloproteinases (MMPs), facilitates extracellular matrix degradation and cell invasion. A number of key EMT transcription factors (EMT‐TFs) have been identified that directly control E‐cadherin expression through transcriptional repression of *CDH1*. These include the SNAIL/SNAI1, SLUG/SNAI2, E47, and zinc finger E‐box‐binding homeobox (ZEB) family of EMT‐TFs, whereas other TFs like TWIST and goosecoid are indirect *CDH1* suppressors (Thiery and Sleeman, 2006). The regulation and activation of the EMT‐TFs is complex involving pleiotropic and contextual signals and different regulatory layers including negative feedback loops with microRNAs (miRNAs) and alterations in the DNA methylation status, facilitating short time or prolonged induction of the mesenchymal state.

Logically, EMT has been particularly studied in carcinomas (epithelial tumors) where it can be locally and time dependently activated to generate tumor cells with enhanced aggressive mesenchymal properties. Much less is known about the role of EMT/MET‐related processes in nonepithelial tumor types such as gliomas, hematopoietic malignancies, and sarcomas. Theoretically, the occurrence of an EMT‐like process in gliomas might be expected considering their origin from primitive epithelium, the neuroectoderm. However, sarcomas and hematological malignancies maintain a mostly mesenchymal status as they originate from muscle or blood cells, respectively, tissues that are derived from the embryonic mesoderm. In this review, we will discuss the current insights into the contribution of EMT/MET‐like processes and underlying mechanisms to the development and progression of nonepithelial tumor types.

## 2 MT in tumors of the central nervous system

During embryonic development before the onset of neurogenesis, the neural plate is formed of a single layer of highly undifferentiated neuroepithelial cells. These neural stem cells (NSC) can differentiate into the three cell types of the brain: neurons, astrocytes, and glial cells. Neurogenesis is also ongoing in distinct areas of the adult brain such as the subventricular zone (SVZ), the olfactory bulb, or the dentate gyrus of the hippocampus (Götz and Huttner, 2005). Although neural tissue does not originate from a classical epithelial background, accumulating evidence indicates that molecular drivers of epithelial cell differentiation take on similar responsibilities in the brain, particularly during tumor development. Below, we summarize the current understanding on how the EMT‐like process is regulated in brain tumors and discuss its utility as a therapeutic and diagnostic target.

### 2.1 Glioblastoma

**Figure 2:**
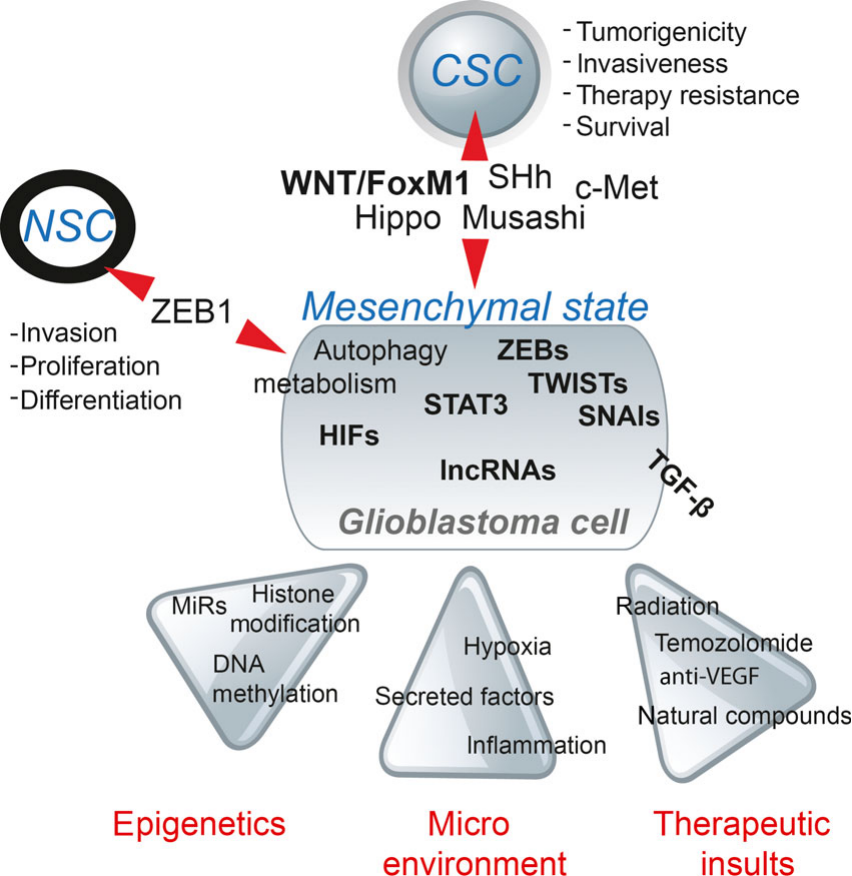
Regulators of MT in glioblastoma. Schematic representation of the complex molecular interactions of MT in glioblastoma including its relationship to cancer stem cells (CSCs), neural stem cells (NSCs). MT can be influenced through epigenetics, microenvironmental stimuli, long noncoding RNAs (lncRNAs) or as a response to therapy.

Glioblastoma (GBM) is the most common and highest malignant primary brain cancer in adults with an average patient survival of less than two years (Stupp *et al*., 2005). GBM, characterized by high invasive growth and high degree of cellular/genetic heterogeneity, accounts to the most lethal tumors overall (Siegel *et al*., 2015). The field of mesenchymal transition (MT) research in GBM has attracted significant attention. The core findings are discussed below and summarized in Fig. 2.

#### 2.1.1 MT in maintenance of neural and GBM stem cells

A tight link between a cellular gain of mesenchymal properties and CSCs has been observed in various tumors including GBM (Liu and Fan, 2015). CSCs are considered to be responsible for tumor occurrence, progression, and emergence of resistance against therapeutic interventions (Lathia *et al*., 2015). Several prominent members of the EMT network have been identified to contribute to the CSC pool in GBMs. For example, ZEB1 promotes the expression of NCS markers and the chemoresistance marker O^6^‐alkylguanine DNA alkyltransferase (MGMT), thereby facilitating GBM tumorigenicity and resistance against temozolomide (TMZ), the standard‐of‐care chemotherapeutic today (Siebzehnrubl *et al*., 2013).

Phylogenetically conserved stem cell signaling pathways represent the most accepted anti‐CSC targets in various tumors including GBM (Kahlert *et al*., 2017; Takebe *et al*., 2015). Interfering with such pathways has been shown to affect MT in GBMs. As such, WNT signaling controls GBM invasion at least in part through the initiation of MT (Bhuvanalakshmi *et al*., 2015; Duan *et al*., 2015; Jin *et al*., 2011; Kahlert *et al*., 2012). Forkhead box M1 (FoxM1) promotes glioma tumorigenicity through the WNT pathway partly by inducing EMT (Wang *et al*., 2015a; Zhang *et al*., 2011). Targeting the Sonic Hedgehog (SHH) pathway at the level of Smoothened suppresses glioma malignancy by upregulating miR200 and consequently blocking ZEB1 (Fu *et al*., 2013). In addition, SHH/glioma‐associated oncogene homolog 1 (Gli1) signaling promotes GBM cell invasion by the induction of EMT‐TF SNAI1 (Wang *et al*., 2010). Recently, Jagged1 ligand‐mediated Notch pathway activation has been shown to promote MT in gliomas (Katz *et al*., 2014). However, currently pharmacological studies that make use of, for example, γ‐secretase inhibitors to further demonstrate the concept that Notch activity controls MT in GBMs are lacking. Furthermore, transcriptional coactivator with PDZ‐binding motif (TAZ), which is primarily regulated through the developmental conserved stem cell pathway Hippo, induces mesenchymal differentiation in GBM and NSC cells and promotes tumor aggressiveness (Bhat *et al*., 2011).

Independent of developmental conserved stem cell pathways, c‐MET signaling induces the reprogramming of glioma cells into CSCs (Li *et al*., 2011). Moreover, c‐MET was recently found to promote MT featuring enhanced MMP activation and cadherin cleavage as well as fostering resistance against TMZ (Huang *et al*., 2016).

The concept that MT in cells of the central nervous system (CNS) is intertwined with stemness is supported by discoveries in developmental biology. ZEB1 expression is high in stem cell‐rich embryonic brain but fades during maturation (Koch *et al*., 2016). ZEB1 controls invasion of human NSC of the SVZ (Kahlert *et al*., 2015), proliferation of spinal cord stem cells of adult mice (Sabourin *et al*., 2009), and restricts differentiation of murine granular neuron precursors (GNP) (Singh *et al*., 2016). Moreover, transforming fetal NSC into invasive tumorigenic cells leads to the induction of an MT signature featuring high SNAI1 expression (Mao *et al*., 2013).

Taken together, several studies have revealed that CSC properties in GBM are linked with the MT network. Targeting MT thus can be an efficient way to eradicate CSCs particularly when interfering with ZEB1 signaling as SNAI1 was reported to play divergent roles in stemness and MT. It is reported that SNAI1 in GBM cells promotes invasion but has a negative effect on tumorigenicity, consistent with the ‘go or grow’ hypothesis (Han *et al*., 2011; Savary *et al*., 2013). The fact that EMT‐TFs also regulate various processes in non‐neoplastic CNS stem cells further supports the existence of a stem cell/MT axis during neuro‐oncogenesis.

#### 2.1.2 Regulators of MT and GBM progression

Several reports have further highlighted the relevance of MT in the progression of GBM. ZEB2 is upregulated in GBM cells and suppression of ZEB2 inhibits invasion (Qi *et al*., 2012) and results in a blockade of resistance against vascular endothelial growth factor (VEGF), which involves inhibition of a hypoxia‐inducible factor (HIF)1a‐ZEB2‐EphrinB2 MT pathway (Depner *et al*., 2016). Similarly, TWIST1 is highly expressed in GBM and is involved in promoting cellular invasion (Elias *et al*., 2005; Mikheeva *et al*., 2010).

The Janus kinases (JAKs) and signal transducer and activator of transcription (STAT) proteins, particularly STAT3, are among the most promising targets for cancer therapy (Yu *et al*., 2014). STAT3 signaling has been identified as a core driver of mesenchymal transdifferentiation in GBMs (Carro *et al*., 2010). STAT3 assists in GBM cell invasion by inducing SNAI1 and activation of MMPs (Priester *et al*., 2013). Recent work suggests that mesenchymal transformation by STAT3 is controlled through Annexin‐A2 (ANXA2) that is inactivated in GBMs that exhibit mutations in the *isocitrate dehydrogenase* (*IDH)* gene (Kling *et al*., 2016).

Interestingly, STAT3 may also suppress the recognition of GBM cells by the immune system (Ciaglia *et al*., 2015). Recent observations suggest that cellular metabolic adaptations such as in choline homeostasis or autophagy can trigger or support the induction of mesenchymal transcription factors in GBM (Koch *et al*., 2016; Zou *et al*., 2016).

Overall, accumulating evidences occur that a variety of potent oncogenic signaling pathways contribute to MT in GBMs.

#### 2.1.3 Tumor microenvironment regulators of MT

The tumor microenvironment (TME), consisting of blood vessels, immune cells, and fibroblasts, is a potent influence for a variety of cellular processes and has been observed to promote mesenchymal transformation in different contexts (Cooper *et al*., 2012). The impact of the TME on MT in GBM is discussed below.

Transforming growth factor beta (TGF‐β), most often produced by immune cells and microglia, is a prominent therapeutic target in GBM and has been investigated both in preclinical and in clinical studies (Joseph *et al*., 2013). Exogenously added TGF‐β induces ZEB1 leading to increased invasiveness, and targeting TGF‐β signaling can block MT in GBMs (Joseph *et al*., 2014). Moreover, in GBM xenografts and patient samples, evidence for local TGF‐β‐dependent MT was provided. In another study, TGF‐β2 transcript levels were demonstrated to serve as a predictive marker for inferior patient overall survival (Frei *et al*., 2015). At the cell surface, the Fas receptor (CD95) has recently been associated with CSC and MT properties in GBM cells, involving activation of PI3K signaling (Drachsler *et al*., 2016).

Shortage of blood supply leads to hypoxia (< 5% oxygen tension) and is a characteristic neuropathological hallmark of GBM. ZEB1 is active in hypoxic pseudopalisades that surround necrotic areas and exposure to low oxygen augments invasive properties of glioma cells through the induction of EMT activators (Depner *et al*., 2016; Joseph *et al*., 2015; Kahlert *et al*., 2015; Xu *et al*., 2015). Given this unequivocal correlation between low oxygen and GBM MT, therapies that aim to block tumor oxygenation such as through blocking VEGF signaling can have contraintended consequences and induce tumor malignancy through the activation of MT (Piao *et al*., 2012). Of note, VEGF signaling has been shown to directly augment GBM cell invasion upstream of MT signaling, and this network is activated in an oxygen‐dependent manner, further raising concerns for the use of anti‐VEGF therapy in GBM. The study by Piao *et al*. also indicates that the activation of the local immune cell environment in response to therapy may promote the mesenchymal shift. Confirming data comes from another study showing that GBM‐associated immune cells produce high levels of TGF‐β, which causes activation of MMPs in the tumor cells to enhance invasion (Ye *et al*., 2012). Given the current rise of immune therapeutic interventions as an innovative treatment avenue in cancer, including GBM, caution needs to be drawn on potential unintended effects such as inducing dissemination of a subset of tumor cells with mesenchymal features.

In conclusion, TME‐derived signals appear to be of key importance in controlling the mesenchymal status of GBM.

#### 2.1.4 MT and response to therapy

Cancer cells are characterized by high plasticity having the ability to adapt to alterations in their microenvironment to overcome cellular stress such as that inflicted by therapeutic treatment. Some indications have been obtained that standard therapies can cause a selection for therapy‐resistant cells that have undergone MT or lead to the initiation of MT. Treatment with radioactive iodine‐125 (^125^I) inhibited ZEB1 and MT accompanied by reduced cellular invasion and growth (Tian *et al*., 2015). GBM cells with an MT signature are less affected by standard treatments. For example, following γ‐radiation of patients SNAI1, EMT markers and invasion were elevated in recurrent GBM samples, and *in vitro*, SNAI1 knockdown prevented radiation‐induced MT leading to reduced cellular invasion (Mahabir *et al*., 2014). Radiation was also found to activate NF‐κB/STAT3 cooperative signaling that was linked to increased expression of intercellular adhesion molecule‐1 (ICAM‐1) and cell invasion (Kesanakurti *et al*., 2013). The standard chemotherapeutic TMZ was reported to select for GBM cells with different properties than the parental cells including an increase in migration and invasion and upregulation of EMT markers like SNAI1 and SNAI2 (Stepanenko *et al*., 2016). As earlier mentioned, treatment with the VEGF inhibitor causes MT in a hypoxia‐dependent manner eventually establishing therapy‐resistant cells (Piao *et al*., 2013). Interestingly, various plant‐derived compounds can also efficiently impair glioma cell invasion through suppression of MT. The spermine derivative kukoamine A causes cytotoxicity and inhibits motility by inducing apoptosis and blocking EMT‐TFs such as SNAI1 (Wang *et al*., 2016). Members of anthocyanidins, a class of polyphenols, have been reported to effectively regulate MT in GBM cells, thereby providing a molecular link on how fruit and vegetable‐rich diet has the potential to fight tumor cell dissemination. As such, delphinidin inhibits TGF‐β signaling to block SNAI1 and invasion of U87 GBM cells (Ouanouki *et al*., 2017). Moreover, periostin, a matricellular protein secreted in the microenvironment, induces MT in GBMs and increases malignant properties such as invasion (Mikheeva *et al*., 2015).

Recent observations in lung cancer suggest that EMT‐dependent tumor cells are preferentially targeted by immune checkpoint antagonists (Chen *et al*., 2014). Also inflammatory stimuli themselves may lead to an induction of cancer cell EMT (Ricciardi *et al*., 2015). Vice versa, the role of EMT in tumor cell immunogenicity is not fully understood and currently controversially discussed (Terry and Chouaib, 2015). However, molecular networks regulating cellular plasticity must be taken into account when designing new immune system‐based therapeutic avenues to fight cancer, including in GBM.

Summarizing, MT induction appears to function as an adaptive response in glioma cells in order to enhance treatment resistance or to facilitate escape from cell toxic niches. Hence, targeting EMT pathways will likely enhance antitumor efficacy of available treatments in GBM.

#### 2.1.5 Epigenetic regulation of MT and long noncoding RNAs

miRNAs are 20‐ to 23‐nucleotide noncoding RNAs that post‐transcriptionally regulate gene activity through RNA silencing. Several miRNAs have been identified as potent targets to impede EMT‐TFs in GBMs. Mostly negative correlations of miRNA expression with tumorigenesis, tumor invasion, and activation of EMT promoters have been described. miR‐7, miR‐21, miR‐23a, miR‐124, miR‐128a/b, miR‐200, and miR‐221 are among the most studied candidates. The field is heavily investigated, and for a concise review about miRNAs regulating MT in GBMs, see Møller *et al*. (2013). Interestingly, recent observations indicate that miRNAs can indirectly influence MT in GBM by modulating their metabolism (Hatziapostolou *et al*., 2013). TMZ treatment was found to enhance autophagy leading to MT that involves regulation by miR‐517c. In glioma cells expressing wild‐type TP53, but not in a TP53‐mutant background, miR‐517c inhibits the activation of autophagy causing a disturbance of the nuclear translocation of TP53 that in turn blocks mesenchymal transformation and autophagy‐induced invasion (Lu *et al*., 2015). Modulating miRNA homeostasis gains attraction as a powerful approach in clinical translation in various diseases and may be a very applicable way to target MT in brain cancer oncology (Li and Rana, 2014).

Alterations in the chromatin packaging density is another epigenetic mechanism to stereologically influence gene activity that is mediated through histone‐modifying enzymes. For example, histone deacetylase 5 (HDAC5) has been shown to promote MT in gliomas and regulate therapy resistance (Liu *et al*., 2015). Of note, HDAC inhibition has emerged as a novel strategy to overcome EMT and chemoresistance in pancreatic cancer (Meidhof *et al*., 2015), but currently little is known whether targeted histone modification has therapeutic potential in GBM.

Promoter methylation is another important mechanism for the control of gene transcription and EMT regulation. Recent evidences occurred showing that expression of ANXA2, an inducer of mesenchymal transformation, is suppressed by promoter hypermethylation and is associated with a better prognosis in CpG hypermethylator phenotype (GCIMP) GBM (Kling *et al*., 2016).

Finally, long noncoding RNAs (lncRNA) have recently emerged as therapeutic targets in various diseases. MT in GBM seems to be influenced by lncRNAs as HOTAIR promotes GBM cell invasion through the activation of WNT‐dependent MT (Zhou *et al*., 2015). LncRNA ZEB1 antisense 1 (ZEB1‐AS1), a noncoding antisense transcript controlled by the ZEB1 promoter, serves as biomarker for poor clinical prognosis in patients with GBM and augments cell invasion by inducing EMT activator ZEB1 (Lv *et al*., 2016). Also, lncRNA AB073614 can influence the expression of mesenchymal differentiation in glioma cells although no effect on EMT‐TFs expression was found (Li *et al*., 2016).

In summary, blocking (E)MT in cancer cells through targeted modification of the epigenome or the household of lncRNAs may be an attractive therapeutic strategy to impede tumor malignancy. This is particularly promising as epigenetic alterations are reversible and may be applied to transiently make tumors more susceptible to traditional, clinically approved drugs. Some promising ongoing clinical trials underline the potential of epigenetic cancer therapy but targeted delivery must be improved to limit off‐target effects (Nervi *et al*., 2015).

#### 2.1.6 Methods for the detection of MT

Liquid biopsy is a minimal invasive procedure for diagnosis and monitoring disease progression. It has recently been identified that circulating brain cancer cells (CBCCs) can be identified in the blood from patients with GBM and those cells are highly tumorigenic as shown in xenotransplantation experiments. Of note, CBCCs exhibit an MT gene signature featuring high *SERPINE1*,* TGFB1*,* TGFBR2*, and *VIM* expression (Sullivan *et al*., 2014). Moreover, noninvasive imaging has emerged as a promising tool in precision medicine. Recently, it was shown that the MT status of GBM cells can be monitored by determining the intracellular composition of choline derivatives using high resolution of proton nuclear magnetic resonance spectroscopy; the targeting of choline kinase 1a (CHK1a) impaired MT (Koch *et al*., 2016).

A classical gene expression analysis is used to predict therapy success. As such, EMT gene expression levels have been found to serve as predictive biomarkers for estimating the overall survival of radiation‐treated GBM patients (Meng *et al*., 2014). Moreover, cellular components excreted in exosomes show promising results to be suitable for diagnosis in oncology. Exosomes have even been found to carry an ‘EMT payload’ such as β‐catenin or HIFs that enhance the invasive and migratory capabilities of recipient cells, thereby indirectly mediating cancer metastasis and cellular dissemination (Syn *et al*., 2016). Interestingly, exosomes of GBM cells were found to reflect the hypoxic signature of the tumors and transmit hypoxia‐associated signals to receiving cells (Kucharzewska *et al*., 2013).

Taken together, we hypothesize that traits of MT in cellular metabolism or spread of proteome/RNA/DNA into the blood or cerebral spinal fluid together with recent technical advantages in noninvasive imaging methods will contribute to the development of highly personalized and minimally invasive diagnostic approaches for patients with cancer extending the importance of (E)MT not only as a therapeutic target but also to diagnostic value.

### 2.2 MT in other cancers of the central nervous system

Although less understood and investigated, several strong indications exist that EMT‐like processes and related factors are involved in the development of other brain malignancies, which will be discussed below.

#### 2.2.1 Medulloblastoma

Medulloblastoma (MB) is the most common primary malignant pediatric brain tumor and can be subcategorized into molecular subgroups (Cho *et al*., 2010). SHH activation in GNP, the believed cellular origin of MBs, as well as in MB cells induces the expression of SNAI1, consequently activating the proto‐oncogene N‐MYC to induce cellular transformation and proliferation (Colvin Wanshura *et al*., 2011). Hypoxia induces MT in MB cells by activating SNAI1, vimentin, and N‐cadherin (Gupta *et al*., 2011). Moreover, ZEB1 expression is high in SHH‐MB and inhibits granular zone exit, which eventually contributes to tumor formation (Singh *et al*., 2016).

#### 2.2.2 Other brain tumor types

A comprehensive histological analysis of ZEB1 in different brain tumors showed that ZEB1 activation correlates with increasing tumor malignancy grade (Kahlert *et al*., 2015). Also assessed by histology, gliosarcomas express high levels of EMT‐TFs such as TWIST1 and SNAI2 (Nagaishi *et al*., 2012). Histone mutations as well as gene expression profiling can differentiate diffuse intrinsic pontine glioma (DIPG), one of the most devastating pediatric brain tumors, into molecular subgroups including a mesenchymal branch (Castel *et al*., 2015; Puget *et al*., 2012). Interestingly, subgrouping according to the expression levels of EMT markers revealed a positive prognostic value, indicating that MT in DIPGs may not play the classical protumorigenic role (Puget *et al*., 2012).

Thus, although not extensively studied as yet, MT appears to play a role in different brain tumor types.

## 3 MT in hematological malignancies

Tumors of the hematopoietic and lymphoid tissues derive from either myeloid or lymphoid blood cell lineages and give rise to leukemias or lymphomas, respectively (Vardiman *et al*., 2009). Although the role of MT in these malignancies originating from the mesoderm is less well studied than for gliomas, there is evidence for the involvement of EMT‐TFs in their malignant progression.

### 3.1 Lymphomas

Lymphomas occur mostly in lymph nodes and can be subdivided in to two major groups, Hodgkin's (HL) and non‐Hodgkin's lymphoma (NHL). NHLs include many different types such as B cell‐derived Burkitt lymphoma, diffuse large B‐cell lymphoma (DLBCL), mantle cell lymphoma (MCL), and T‐cell lymphomas (Sun *et al*., 2016).

#### 3.1.1 MT inducers in B‐cell malignancies

A role for ZEB1 in the regulation of *B‐cell lymphoma protein 6* (*BCL6*), a master transcription factor in the differentiation and development of B cells, has been reported (Papadopoulou *et al*., 2010). ZEB1 together with C‐terminal binding protein binds to the *BCL6* promoter leading to transcriptional suppression and contribute to normal B‐cell differentiation and development. In malignancies, BCL6 expression in DLBCL has been linked to better prognosis, and consistent with this, immunohistochemical analyses of diagnostic patient samples indicated a correlation between nuclear ZEB1 staining and adverse clinical presentation and clinical outcome (Lemma *et al*., 2013). In *Helicobacter pylori*‐positive gastric DLBCL, which has a better prognosis than negative counterparts, also a role for ZEB1 has been implicated. Molecular analysis revealed an association between elevated expression of miR‐200 and consequently inhibition of ZEB1 and an increase in BCL6 expression in *H. pylori*‐positive samples (Huang *et al*., 2014). This provides a further link between ZEB1 expression and a more aggressive DLBC phenotype. Involvement of miR‐200 also has been described in a radiation‐induced thymic lymphoma (RITL) mouse model. RITL samples showed a decrease in miR‐200c and forced expression of this miRNA resulted in cell death, which was associated with suppression of the polycomb group protein BMI1 (Cui *et al*., 2014).

In MCL, also an important role for ZEB1 in tumor aggressiveness has been identified. MCL is a rare B‐cell malignancy demonstrating resistance to treatment and poor prognoses. In half of the MCL cases, the canonical WNT pathway is activated, and recently, this was linked to ZEB1 expression and a shorter overall survival (Sanchez‐Tillo *et al*., 2014). Beta‐catenin could bind and activate the *ZEB1* promoter and ZEB1 protein on its turn activates proliferation and antiapoptotic genes while suppressing proapoptotic ones. Moreover, ZEB1 contributes to chemoresistance by enhancing expression of drug efflux pathways and consequently silencing of ZEB1, resulting in sensitization to doxorubicin in a xenograft mouse model.

#### 3.1.2 MT inducers in T‐cell lymphomas

Sézary syndrome (Sz) is a rare cutaneous T‐cell lymphoma (CTCL) that primarily manifests in the skin. Analyses of the T cells in Sz patients and normal controls revealed particularly high selective expression of EphA4 and TWIST as well as in other types of CTCL (van Doorn *et al*., 2004). More recently, promoter DNA hypomethylation of TWIST has been associated with protein overexpression (Wong *et al*., 2015).

In addition to TWIST, also ZEB1 has been suggested to be involved in Sz CTCL. Genomic analyses revealed *ZEB1* gene deletions in more than half of the cases although the relevance of this deletion needs to be further explored (Wang *et al*., 2015b). Recently, a possible mechanism was provided. Biopsies of patients with CTCL demonstrated increased interleukin (IL)‐15 activity that appears instrumental for disease progression. Interestingly, ZEB1 has been identified as a potent transcriptional repressor of *IL‐15* and hypermethylation of its binding region in the *IL‐15* promoter prevents suppression of IL‐15 production in CTCL and thus progression (Mishra *et al*., 2016).

A chromosomal translocation giving rise to an abnormal nucleophosmin (NPM)‐anaplastic lymphoma kinase (ALK) fusion protein is characteristic for pediatric anaplastic large cell lymphoma (ALCL). TWIST1 was found aberrantly expressed in ALK + ALCL cells, which could be attributed to constitutive STAT3 signaling in this T‐cell malignancy (Zhang *et al*., 2012). TWIST1 knockdown decreased invasiveness and sensitized for an ALK inhibitor in cell culture models, thus linking TWIST1 with malignant progression and therapy resistance of this tumor.

Binding of ZEB1 to SMAD3 and SMAD7 enhances TGF‐β signaling. In accordance with that, downregulation of ZEB1 resulted in resistance to the growth‐suppressive effect of TGF‐β in adult T‐cell leukemia/lymphoma (ATLL) (Nakahata *et al*., 2010). In this context, it is interesting to note that ZEB1 is involved in the regulation of normal T‐cell development. Mice with homozygous C‐terminal deletions in *Zeb1* were among others characterized by small thymus and a reduction in early T‐cell progenitors (Higashi *et al*., 1997). This provides another hint that ZEB1 is an important regulator of differentiation pathways that are deregulated in lymphoid malignancies.

### 3.2 Myelomas

Multiple myeloma (MM) originates from plasma cells that normally produce antibodies. It is the second most common hematological malignancy that still remains incurable. Hypoxia, a well‐known trigger of EMT in solid tumors, also is able to induce this process in MM cells. Hypoxia in bone marrow (BM) niches resulted in MT in MM cells characterized by a decrease in E‐cadherin levels and increases in EMT‐inducing proteins such as SNAI1 and TGF‐β, which positively correlated with levels of circulating MM cells in the peripheral blood. In addition, hypoxia‐induced CXCR4 resulted in homing of MM cells to the BM, thus completing a malignant dissemination–colonization cycle (Azab *et al*., 2012). The involvement of MT in disease spreading is also supported by the finding that TWIST1 expression is elevated in skeletal extramedullary disease of patients with MM and correlates with a lower rate of progression‐free survival (Yang *et al*., 2016). In another study, IL‐17 is reported to enhance cell proliferation and repress cell adhesion by inducing MT evidenced by downregulation of E‐cadherin and upregulation of SNAI1, SNAI2, and vimentin. In addition, IL‐17 repressed miR‐192 that targets the IL‐17 receptor, thus providing a regulatory feedback loop (Sun *et al*., 2014). Based on these findings, it is postulated that inhibition of MT may provide therapeutic benefit in MM.

### 3.3 Leukemias

The involvement of *TWIST2* in leukemia was first described by Raval *et al*. (2005) in chronic lymphocytic leukemia (CLL). Absence of TWIST2 expression correlated with *TWIST2* promoter methylation in a proportion of CLL cases. The epigenetic inactivation of *TWIST2* also has been reported to modulate disease progression in childhood acute lymphoblastic leukemia. Promoter methylation of *TWIST2* was found in more than half of the cases and restoration of TWIST2 expression resulted in growth inhibition and apoptosis *in vitro* suggestive of tumor‐suppressive functions (Thathia *et al*., 2012). Consistent with this notion is a report by Zhang *et al*. (2015), showing that in acute myeloid leukemia (AML) in around 30% of examined cases hypermethylation of *TWIST2* occurred leading to reduced expression of both TWIST2 and the cyclin‐dependent kinase inhibitor p21. TWIST2 activates p21 expression among other tumor suppressor genes and suppresses oncogenic activity.

In contrast to TWIST2, TWIST1 was linked with enhanced aggressiveness of leukemic cells. In chronic myeloid leukemia (CML), upregulation of TWIST1 was seen in patient samples obtained from imatinib‐resistant patients. A link between imatinib resistance and TWIST1 exists, as *in vitro* knockdown of TWIST1 expression in CML cells resulted in sensitization for imatinib (Cosset *et al*., 2011). TWIST1 also has been identified as a direct regulator of *BMI*, which is known for its role in maintaining self‐renewal, a characteristic of cells with a high proliferative potential or stem cells. Analyses of TWIST1 and BMI expression in AML revealed a positive correlation associated with enhanced proliferation and apoptosis resistance *in vitro* (Chen *et al*., 2015). In addition, enhanced TWIST1 expression was found in CML leukemic stem cells that decreased upon differentiation. Downmodulation of TWIST1 reduced their colony‐forming capacity, providing further evidence for TWIST1 involvement in leukemia stem cells and disease progression (Wang *et al*., 2015c).

Recently, using an inducible *MLL‐AF9*‐driven AML mouse model representing an aggressive type of AML, elevated expression of EMT‐related genes has been observed. Knockdown of ZEB1 reduced the invasive properties of this aggressive tumor (Stavropoulou *et al*., 2016). In another study, shRNA screens to identify genetic dependencies for AML resulted in the identification of ZEB2. ZEB2 downregulation impaired proliferation and caused irregular differentiation of AML cells (Li *et al*., 2017). The implications of MT in leukemia also is demonstrated by a report showing that an increase in HIF1‐related signaling is associated with genes involved in EMT, further linking EMT‐like processes with leukemia progression (Percio *et al*., 2014).

Together, the above illustrates that EMT inducers and EMT‐TFs play important roles in the progression of hematological tumors.

## 4 EMT‐ and MET‐related processes in sarcomas

Sarcomas are uncommon malignancies that arise from mesenchymal cell types and develop in or from bone, cartilage, or connective tissue, such as muscle, fat, peripheral nerves, and fibrous or related tissues (Taylor *et al*., 2011). Together, sarcomas account for nearly 21% of all pediatric solid cancers and less than 1% of all adult solid malignant cancers (Surveillance, Epidemiology, and End Results (SEER) program (Burningham *et al*., 2012). Sarcomas can be predominantly grouped into two major groups, namely malignant bone tumors and soft tissue sarcomas (Lahat *et al*., 2008).

**Figure 3:**
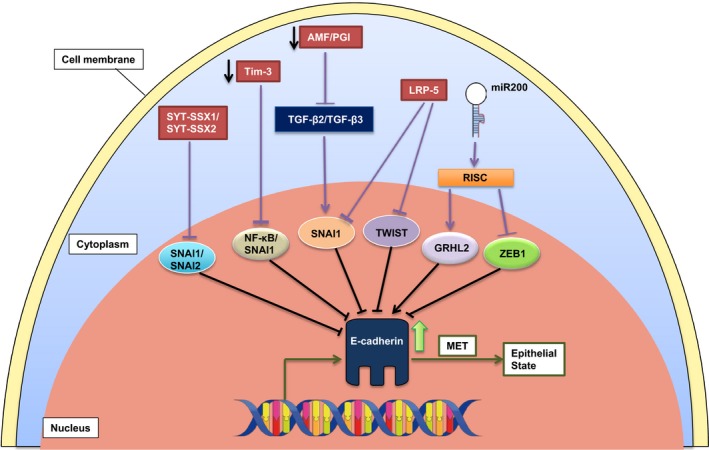
Initiation and regulation of MET in sarcomas. Summary of mechanisms identified in different sarcoma subtypes (see also text). Upstream signaling involving the oncogenic fusion proteins SYT‐SSX1 and SYT‐SSX2 downregulates SNAI1 or SNAI2 leading to an increase in E‐cadherin expression, a readout for MET. Downregulation of Tim‐3 leads to epithelial differentiation via suppressing NF‐κB/SNAI1 signaling. The silencing of the AMF/PG1 complex results in enhanced secretion of TGF‐β2 and TGF‐β3, which in turn can induce SNAI1 giving way to an elevated E‐cadherin expression. LRP5, a component of WNT signaling, can upregulate E‐cadherin, which is brought about by the suppression of SNAI2 and TWIST. mir200 exerts a dual effect as its overexpression can lead to GRHL2 overexpression and downregulation of ZEB1 resulting in elevated E‐cadherin levels. Together, all these signals mostly lead to an elevation in E‐cadherin and consequently MET, which has been linked with a better patient prognosis.

As sarcomas are mesenchymal by default, some studies have addressed whether EMT inducers are involved in tumorigenesis. Indeed, SNAI1 expression is associated with worse overall survival in sarcomas. In addition, ectopic expression of SNAI1 has tumorigenic activity in fibroblasts and a role for SNAI1 in the generation of sarcomas has been suggested (Alba‐Castellón *et al*., 2014). Similarly, in malignant bone tumors, osteosarcomas, elevated levels of ZEB1 are detected compared to normal bone and ZEB1 expression is higher in metastatic osteosarcoma in comparison with the group without metastases (Shen *et al*., 2012). A recent study shows that TGF‐β treatment can trigger MT of osteosarcoma cells *in vitro* involving estrogen‐related receptor α‐dependent activation of SNAI1 (Chen *et al*., 2016). Thus, MT may be involved in the onset and progression of sarcomas but more studies are required for further substantiation. However, most studies have focused on studying the involvement of the reverse process, MET, in malignancy of sarcomas (see also Fig. 3).

### 4.1 Epithelial differentiation and prognosis in sarcomas

A number of studies have reported the occurrence of local epithelial differentiation in sarcomas by determining the expression of epithelial markers. Sato and coworkers were one of the first showing E‐cadherin expression in various bone and soft tissue sarcomas (Sato *et al*., 1999). Likewise, in synovial sarcomas, the expression of epithelial markers like E‐cadherin and β‐catenin was detected and a decrease in their expression was linked to a high potential of recurrence or metastasis and poor prognosis (Saito *et al*., 2004, 2006). Ewing sarcoma/primitive neuroectodermal tumor, a primitive bone and soft tissue sarcoma, frequently displays partial epithelial differentiation evidenced by the expression of tight junction proteins claudin‐1 and ZO‐1, although being negative for E‐cadherin expression (Schuetz *et al*., 2005). In osteosarcomas, expression of E‐cadherin is potentially useful as a prognostic marker for patient survival (Nakajima *et al*., 2008). An MET transcription profile appeared prognostic for improved survival in sarcoma patients (Yang *et al*., 2010). This is in agreement with another study showing that sarcoma patients with higher levels of the epithelial marker, E‐cadherin, have improved survival in comparison with those with low or no E‐cadherin (Wang *et al*., 2015d). It should be noted that despite the increase in epithelial markers in sarcomas, mesenchymal markers continue to be abundantly expressed (Saito *et al*., 2004; Yang *et al*., 2010).

Evidence is growing for the utilization of miRNA profiling in the diagnosis of soft tissue sarcomas (Fujiwara *et al*., 2014). As miRNAs are known for regulating EMT/MET processes, it is likely that they affect the mesenchymal status of sarcomas. Only few studies have explored this (see under 4.2) and greater understanding of the biology of miRNAs in sarcomas will undoubtedly contribute the advancement of novel diagnostic and therapeutic approaches.

### 4.2 Mechanisms of MET in sarcomas

Additional evidence for the occurrence of MET in sarcomas has been obtained by functional studies aiming at elucidation of the underlying mechanisms of epithelial differentiation. In synovial sarcoma, the fusion proteins SYT‐SSX1 and SYT‐SSX2 are able to interact with SNAI1 or SNAI2, thus preventing their suppressive effects on E‐cadherin expression leading to the acquisition of epithelial features indicative of MET (Saito *et al*., 2006). An integrated proteomics and genomics analyses in soft tissue leiomyosarcomas identified SNAI2/SLUG as a negative regulator of E‐cadherin expression; knockdown of SNAI2 increased E‐cadherin and decreased vimentin expression that was associated with a decrease in proliferation and invasion (Yang *et al*., 2010). A more recent study demonstrates that the combined expression of miR‐200 family members and upregulation of an epithelial gene activator, grainyhead‐like transcription factor 2 (GRHL2), drive MET in sarcomas. This study showed that both GRHL2 overexpression and downregulation of ZEB1 by either RNAi‐mediated silencing or miR‐200 overexpression act in a synergistic manner to control the upregulation of epithelial genes, including E‐cadherin, and consequently MET (Somarelli *et al*., 2016). MET‐like phenomena also have been detected in chondrosarcomas where a downregulation of SNAI1/SNAIL led to a gain of mesenchymal markers like E‐cadherin, desmocollin, maspin, and 14‐3‐3σ that in part were regulated epigenetically by cytosine methylation (Fitzgerald *et al*., 2011). Yet another study demonstrated that the expression of T‐cell immunoglobulin mucin domain molecule‐3 (Tim‐3) in osteosarcomas contributes to the mesenchymal status. Tim‐3 downregulation significantly suppressed osteosarcoma cell (MG‐63) proliferation and metastasis via inhibition of the NF‐κB/SNAI1 signaling pathway causing epithelial differentiation (Feng and Guo, 2016).

Phosphoglucose isomerase (PGI), a glycolytic enzyme catalyzing an interconversion between glucose and fructose, extracellularly behaves as a cytokine that includes autocrine motility factor (AMF). AMF/PGI has been typically associated with induction of EMT. It has been shown in osteosarcoma that the silencing of AMF/PGI reduces the production and secretion of TGF‐β2 and TGF‐β3 resulting in downregulation of SNAI1 that can elevate E‐cadherin expression leading to MET. Thus, silencing of AMF/PGI might contribute toward the loss of malignancy in these cancers through differentiation via MET (Niinaka *et al*., 2010). Similar to this study, AMF/PGI appears to regulate the MET process in human lung fibrosarcoma cells (Funasaka *et al*., 2007). In Saos‐2 osteosarcoma cells, transfection of WNT receptor low‐density lipoprotein receptor‐related protein 5 (LRP5) caused a marked upregulation of E‐cadherin and downregulation of N‐cadherin and was associated with reduced activity of the transcription factors SNAI2 and Twist (Guo *et al*., 2007). A detailed overview of the upstream signaling and transcription factors involved in the initiation and regulation of MET in sarcoma is depicted in Fig. 3.

Together, these findings indicate the involvement of the well‐known EMT inducers in either the maintenance of mesenchymal differentiation or the onset of epithelial differentiation upon loss of activity, thus explaining the observed heterogeneity in this mesenchymal neoplasm.

### 4.3 MT/MET and therapy in sarcomas

Several studies have implicated the involvement of MT and MET in sensitivity of sarcomas to therapies. The exposure of osteosarcoma cells to cisplatin generates a more resistant and mesenchymal phenotype as was shown in *in vitro* and *in vivo* studies. SNAI1 appeared to be the major factor mediating this cisplatin‐induced MT (Fang *et al*., 2016). The use of radiosensitizers like zoledronic acid can sensitize osteosarcoma cells to γ‐irradiation. Sensitization is associated with impaired cell migration, invasion and reduced expression of EMT markers like vimentin, MMP‐9, and SNAI2, indicating epithelial differentiation and reduced malignancy (Kim *et al*., 2016). An interesting drug against sarcomas is the naturally derived bioactive compound withaferin‐A (WFA) that targets vimentin. WFA induces marked apoptosis and vimentin cleavage in vimentin‐expressing tumor cells. In a cell panel representing different types of sarcoma, high sensitivity to WFA was observed linked to caspase‐dependent degradation of vimentin and apoptosis activation. The proapoptotic response was suppressed following vimentin knockdown or by caspase blockade. WFA also significantly blocked soft tissue sarcoma growth, local recurrence, and metastasis in xenograft models (Lahat *et al*., 2010). This finding holds great promise for the use of WFA and other antivimentin drugs as a potential therapeutic option in soft tissue sarcomas.

## 5 Concluding remarks

Increasing evidences occur that similar, well‐coordinated processes of EMT and its counterpart MET extend to the biology of nonepithelial malignancies. In brain tumors, hematopoietic tumors, and sarcomas, EMT‐like processes also contribute to malignancy, as outlined above. In these nonepithelial cancers, MT is controlled, equally as found in carcinomas, by comparable cell autonomous or TME‐derived signals leading to the modulation of the well‐known EMT‐TFs. Although not as extensively investigated for all nonepithelial tumors and best in GBM, overall MT induction is associated with increased stem cell and invasive/metastatic potential and therapy resistance. Correspondingly, biomarkers for MT have been linked with worse clinical outcome in these cancers. However, particularly in sarcomas, the opposite process MET occurs frequently resulting in more favorable tumor properties. A therapeutic shifting of sarcomas to a more epithelial‐like state could attenuate their aggressiveness and improve patient outcome. Given the fact that recent observations suggest cancer EMT can be monitored through noninvasive or minimal invasive technologies, it will be interesting to correlate the preclinical observations with clinical cohorts and determine possible clinical implications.

Recent observations suggest that the distinct balance between EMT and MET in the form of a hybrid epithelial/mesenchymal (E/M) cellular phenotype particularly promotes cancer cell aggressiveness, which is more drastic than fully committed EMT (Jolly *et al*., 2016). Importantly, the existence of E/M condition(s) in cells of tumors originating from tissues other than the epithelial layer has not been studied comprehensively. Studies to investigate the intermediate stage of E/M in those tumor types are needed to verify whether such a reciprocal, triple phenotypic classification exists and has similar tumor progressive properties as for epithelial cancers.

Given the complexity of EMT/MET networks and the ability of cancer cells to adapt to stress situations, targeting one protein or pathway may not be sufficient to completely impede EMT. Anti‐EMT therapy should therefore be supported by other (targeted) therapies.

In conclusion, further studies are required to unravel the mechanisms governing MT/MET in these nonepithelial cancers and for being suitable as prognostic markers or therapeutic targets in order to improve their clinical management.

## Author contributions

UK, JJ, and FK conceived and wrote the manuscript. FK finalized the manuscript.
